# Molecular Surveillance of Viral Genotypes and Aerobic Bacterial Coinfections in Respiratory Infections: Insights From a Tertiary Care Setting in Iran

**DOI:** 10.1155/bmri/1239885

**Published:** 2026-09-09

**Authors:** Saeed Javdan, Sharareh Moghim, Farzaneh Mohammadzadeh Rostami, Kiana Shirani, Elahe Nasri, Bahram Nasr Esfahani

**Affiliations:** ^1^ Department of Bacteriology and Virology, School of Medicine, Isfahan University of Medical Sciences, Isfahan, Iran, mui.ac.ir; ^2^ Antimicrobial Resistance Research Center, Communicable Diseases Institute, Mazandaran University of Medical Sciences, Sari, Iran, mazums.ac.ir; ^3^ Nosocomial Infection Research Center, Isfahan University of Medical Sciences, Isfahan, Iran, mui.ac.ir; ^4^ Department of Milad Hospital, Isfahan University of Medical Sciences, Isfahan, Iran, mui.ac.ir

**Keywords:** antimicrobial resistance, bacterial coinfection, molecular surveillance, SARS-CoV-2, viral respiratory infections

## Abstract

**Introduction:**

Secondary bacterial infections play a pivotal role in the progression and outcomes of severe viral respiratory diseases. Comprehensive surveillance of both viral genotypes and bacterial pathogens is essential for guiding antimicrobial stewardship, reducing inappropriate antibiotic prescriptions, and preventing the rise of multidrug‐resistant organisms.

**Methods:**

Endotracheal aspirate (ETA) samples were collected from 150 patients admitted with viral respiratory infections, including SARS‐CoV‐2 cases. Bacterial isolates were identified through conventional and molecular techniques, and their antibiotic susceptibility profiles were determined according to Clinical and Laboratory Standards Institute (CLSI) guidelines. Viral genotyping was performed by sequencing the spike region.

**Results:**

Among 150 patients, 135 (90%) were confirmed as SARS‐CoV‐2 positive. Secondary bacterial respiratory infections were detected in 53 cases (39.2%). Predominant isolates included *Klebsiella pneumoniae* (28.3%, *n* = 15), *Acinetobacter baumannii* (18.9%, *n* = 10), *Escherichia coli* (15.1%, *n* = 8), *Pseudomonas aeruginosa* (9.4%, *n* = 5), *Staphylococcus aureus* (9.4%, *n* = 5), *Shigella* spp. (5.7%, *n* = 3), *Burkholderia* spp. (3.8%, *n* = 2), along with fungal infections (9.4%, *n* = 5). High resistance rates were observed in *A. baumannii* and *K. pneumoniae*, whereas *E. coli* and *S. aureus* showed greater sensitivity. Viral sequencing identified Alpha (B.1.1.7, 30.2%) and Delta (B.1.617.2, 69.8%) variants among SARS‐CoV‐2 cases.

**Discussion and Conclusion:**

Findings highlight the importance of dual surveillance for viral genotypes and bacterial coinfections in respiratory diseases. Antimicrobial resistance among gram‐negative pathogens poses a major clinical threat, underscoring the need for routine susceptibility testing. Integration of molecular virology with bacteriology not only improves patient outcomes during epidemics such as COVID‐19, but also informs long‐term strategies against emerging pathogens and resistant strains.

## 1. Introduction

Respiratory viral infections such as influenza, respiratory syncytial virus (RSV), and coronaviruses are among the leading global causes of morbidity and mortality. A key factor aggravating disease severity is the occurrence of secondary bacterial infections, which prolong hospital stays, increase the need for intensive care, and contribute substantially to mortality [1]. In parallel, the widespread empirical use of antibiotics in patients with viral infections has accelerated the global crisis of antimicrobial resistance (AMR), underscoring the importance of accurate diagnosis and evidence‐based antimicrobial stewardship [1, 2]. The mechanisms underlying viral–bacterial interactions are multifactorial. Viral infections alter epithelial integrity, immune signaling, and mucosal microbiota composition, creating conditions favorable to bacterial colonization and invasion [3]. Dysbiosis of the respiratory tract microbiome further compromises host defenses and influences susceptibility to opportunistic pathogens such as *Klebsiella pneumoniae*, *Acinetobacter baumannii*, and methicillin‐resistant *Staphylococcus aureus* (MRSA). These pathogens not only complicate clinical management but also exhibit alarming levels of AMR [4, 5]. Genomic surveillance of viral pathogens provides additional insights into disease outcomes and public health risks. Variability in viral genomes, particularly in structural proteins involved in host interaction and immune evasion, can modulate clinical presentation and influence patterns of bacterial coinfection [6]. Thus, an integrated approach that combines molecular virology with bacteriology is critical for improving patient management and informing antimicrobial policies [7]. Despite growing evidence of bacterial coinfections in viral respiratory diseases, most studies have focused either on the bacterial pathogens or the viral etiology in isolation [8]. Consequently, the relationship between specific viral genotypes and the pattern of secondary bacterial infections remains poorly understood [9]. For instance, emerging SARS‐CoV‐2 variants have been shown to differ in their pathogenicity, immune evasion capacity, and clinical outcomes, but whether they differentially predispose patients to bacterial superinfections has not been systematically investigated [10, 11].

Given these considerations, this study is aimed at investigating the prevalence and AMR patterns of aerobic bacterial pathogens among patients with viral respiratory infections in tertiary care hospitals in Isfahan, Iran. In parallel, we examined the genomic profiles of circulating viral variants, using SARS‐CoV‐2 as a case study, to explore possible associations between viral genotype, bacterial coinfection, and resistance patterns. The findings are expected to contribute to broader strategies for integrated surveillance and antimicrobial stewardship in the context of both current and future respiratory epidemics.

## 2. Materials and Methods

### 2.1. Study Participants and Initial Laboratory Assessments

This cross‐sectional study included 150 COVID‐19 patients admitted to ICUs in Isfahan (June 2022–February 2023). Patients had not received antimicrobial therapy for ≥ 1 month prior. Ethical approval was obtained from the Institutional Review Board of Isfahan University of Medical Sciences (IR.MUI.MED.REC.1400.839). The need for informed consent was waived by the ethics committee since this study utilized anonymized residual specimens and retrospective laboratory data without any participant identifiers. All patient data were deidentified prior to analysis, and access to the study database was restricted to the research team. Clinical trial registration was not applicable. Laboratory parameters including complete blood count (CBC), erythrocyte sedimentation rate (ESR), arterial blood gas (ABG) analysis, lactate dehydrogenase (LDH), and C‐reactive protein (CRP) were extracted from the patients′ medical records. Serial monitoring of these values was performed in accordance with each patient’s clinical course, and all data were entered into a dedicated, study‐specific case report form.

We recognize that the lack of a contemporaneous non‐COVID‐19 control cohort restricts the broader applicability of our observations to other viral respiratory pathogens; consequently, the present findings should be regarded as specific to the COVID‐19 population examined in this investigation.

### 2.2. Sample Preparation and Bacterial Isolation

Endotracheal aspirate (ETA) specimens were aseptically collected into sterile containers according to standard clinical protocols [12] and promptly transported to the microbiology laboratory. Samples were inoculated onto blood agar, chocolate agar, eosin methylene blue (EMB) agar, and MacConkey agar, and incubated aerobically at 37°C for 24–72 h. Isolated colonies were identified by Gram staining and conventional biochemical tests, including catalase, oxidase, mannitol salt agar, novobiocin susceptibility, and the API 10S system (ZIPAS, Iran). The biochemical reactions assessed were indole (IND), ONPG, citrate (CIT), glucose (GLU), lysine decarboxylase (LDC), tryptophan deaminase (TDA), arabinose (ARA), urease (URE), ornithine decarboxylase (ODC), H_2_S production, and nitrate reduction (NO_2_) [13, 14].

### 2.3. Molecular Identification of Strains

Bacterial genomic DNA was extracted from 1.5 mL of culture. Cells were harvested by centrifugation (12,000 rpm, 3 min), resuspended in 200 *μ*L lysis buffer (40 mM Tris‐acetate pH 7.8, 20 mM sodium acetate, 1 mM EDTA, 1% SDS), and lysed by repeated pipetting. After addition of 50 *μ*L 5 M NaCl and centrifugation (12,000 rpm, 10 min, 4°C), the supernatant was extracted with an equal volume of chloroform. DNA was precipitated with absolute ethanol, washed twice with 70% ethanol, air‐dried, and resuspended in TE buffer [15]. A fragment of the 16S rRNA gene was amplified using the universal primers F (5 ^′^‐CCAGCAGCCGCGGTAATACG‐3 ^′^) and R (5 ^′^‐ATCGG(C/T)TACCTTGTTACGACTTC‐3 ^′^) with 2 *μ*L of extracted DNA as template [16]. PCR was performed using a commercial master mix (Ampliqon, Odense, Denmark) according to the manufacturer′s instructions. Each 25 *μ*L reaction contained 12 *μ*L master mix, 0.6 *μ*L of each primer (10 pmol/*μ*L), 2 *μ*L template DNA, and 10.4 *μ*L nuclease‐free water. Amplification was carried out on a T100 thermal cycler (Bio‐Rad) with the following conditions: initial denaturation at 94°C for 5 min; 30 cycles of 94°C for 30 s, 45°C for 1 min, and 65°C for 8 min; and a final extension at 65°C for 16 min [17]. Reference strains (*S. aureus* ATCC 25923, *S. epidermidis* ATCC 12228, *E. coli* ATCC 25922, *K. pneumoniae* ATCC 13883, and *P. aeruginosa* ATCC 27853; Iranian Biological Resource Center) were included as controls. Amplicons were separated on 1.5% agarose gels in 1× TBE buffer (65 V, 2 h), sequenced, and identified to the genus and species level by BLAST analysis against the NCBI database. Sequence identity was further verified by high similarity scores and phylogenetic reconstruction using MEGA software.

### 2.4. Antimicrobial Susceptibility Testing

Antimicrobial susceptibility testing was performed by the disk‐diffusion method on Mueller–Hinton agar in accordance with Clinical and Laboratory Standards Institute (CLSI) guidelines [18]. The following agents were tested according to gram‐positive or gram‐negative isolate identity: cefepime (30 *μ*g), cefoxitin (30 *μ*g), imipenem (10 *μ*g), ceftazidime (30 *μ*g), amikacin (30 *μ*g), meropenem (10 *μ*g), azithromycin (15 *μ*g), gentamicin (10 *μ*g), tetracycline (30 *μ*g), ceftriaxone (30 *μ*g), ciprofloxacin (5 *μ*g), trimethoprim–sulfamethoxazole (1.25/23.75 *μ*g), levofloxacin (5 *μ*g), penicillin (10 U), erythromycin (15 *μ*g), piperacillin–tazobactam (100/10 *μ*g), ampicillin–sulbactam (10/10 *μ*g), and cefotaxime (30 *μ*g).

### 2.5. Viral Genotyping

Viral RNA was extracted from ETA specimens using the LabPrep Viral DNA/RNA Mini Kit according to the manufacturer′s instructions, followed by cDNA synthesis. A region of the SARS‐CoV‐2 spike gene was amplified by real‐time PCR using the primers F (5 ^′^‐CCCTGTTGCTATTCATGCAG‐3 ^′^) and R (5 ^′^‐CCCTATTAAACAGCCTGCAC‐3 ^′^) [19]. Amplicons from 53 samples were subjected to bidirectional Sanger sequencing (Macrogen, Seoul, Korea). Sequence data were analyzed with BLAST, ChromasPro 2, and MEGA 11 to assign viral lineages.

## 3. Results

**Figure 1:**
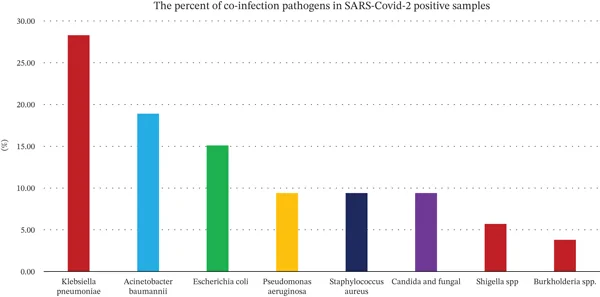
Distribution of respiratory pathogens with the SARS‐CoV‐2 coinfection.

Among 150 patients hospitalized with severe viral respiratory infections, 135 (90%) were confirmed as SARS‐CoV‐2 positive by PCR. Of these, 81 (60%) were male and 54 (40%) female. Secondary bacterial infections were detected in 53 cases (39.2%), with *K. pneumoniae* (28.3%) and *A. baumannii* (18.9%) as the predominant pathogens, whereas *Burkholderia* spp. (3.8%) represented the least frequent isolate (Figure 1). Although the primary focus of this study was on bacterial coinfections, we also observed a small number of fungal coinfections (9.4%, *n* = 5), which are reported here as incidental findings given their clinical relevance in critically ill ICU patients.

**Figure 2:**
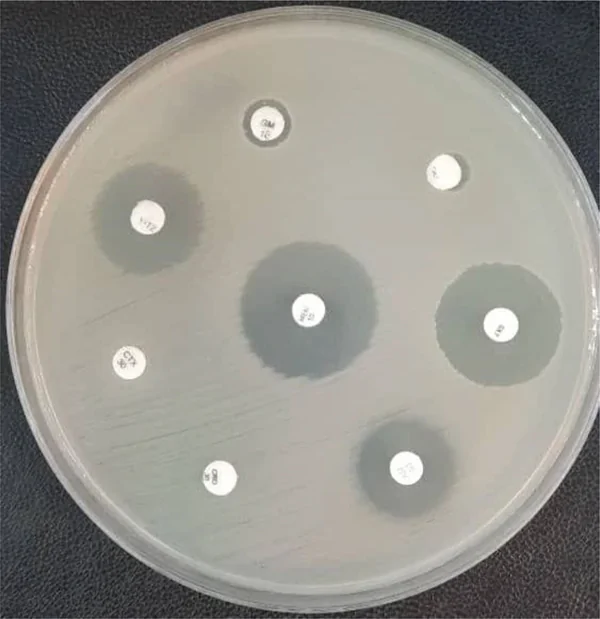
Representative disk diffusion antimicrobial susceptibility testing result. Susceptibility patterns are interpreted based on CLSI guidelines.

Antimicrobial susceptibility testing revealed alarming resistance rates among nonfermentative gram‐negative bacilli. *A. baumannii* exhibited universal resistance to *β*‐lactams, cephalosporins, and carbapenems, with marginal sensitivity to aminoglycosides (≤ 10%). *P. aeruginosa* isolates similarly displayed extensive resistance, with amikacin (40%) as the only effective agent. Conversely, fermentative organisms such as *E. coli* showed overall low resistance (≤ 12.5%). Among gram‐positives, *S. aureus* isolates were universally resistant to penicillin, with 50% classified as MRSA, yet remained fully susceptible to macrolides and tetracyclines (Table 1), (Figure 2).

**Table 1:** The antibiotic sensitivity patterns of bacterial isolates.

Antibiotic sensitivity patterns			Bacterial isolates						
			*S. aureus*	*E. coli*	*K. pneumoniae*	*Shigella*	*Burkholderia*	*Acinetobacter*	*P. aeruginosa*
Penicillin	P	S	0	—	—	—	—	—	—
		I	0						
		R	100%						
	FOX	S	50%	—	—	—	—	—	—
		I	0						
		R	50%						
Macrolides	AZM	S	100%	—	—	—	—	—	—
		I	0						
		R	0						
	E	S	100%	—	—	—	—	—	—
		I	0						
		R	0						
Folate pathway antagonists	SXT	S	75%	87.5%	13.3%	0	100%	10%	20%
		I	0	0	0	0	0	0	0
		R	25%	12.5%	86.67%	100%	0	90%	80%
Tetracyclines	TE	S	100%	—	—	—	—	—	—
		I	0						
		R	0						
Fluroquinolones	CP	S	100%	87.5%	6.67%	0	50%	0	0
		I	0	0	0	33.34%	50%	0	0
		R	0	12.5%	93.33%	66.66%	0	100%	100%
	LEV	S	100%	87.5%	6.67%	0	100%	0	0
		I	0	0	0	0	0	0	0
		R	0	12.5%	93.33%	100%	0	100%	100%
Aminoglycosides	Gm	S	100%	87.5%	6.67%	66.66%	0	10%	0
		I	0	0	0	0	0	10%	0
		R	0	12.5%	93.33%	33.34%	100%	80%	100%
	Ak	S	—	87.5%	40%	66.66%	0	10%	40%
		I		0	13.34%	0	0	0	0
		R		12.5%	46.66%	33.34%	100%	90%	60%
Cephems	CAZ	S	—	87.5%	6.67%	33.34%	0	0	0
		I		0	0	0	0	0	0
		R		12.5%	93.33%	66.66%	100%	100%	100%
	CTX	S	—	87.5%	6.67%	0	0	0	0
		I		0	0	0	50%	0	0
		R		12.5%	93.33%	100%	50%	100%	100%
	CRO	S	—	87.5%	6.67%	0	50%	0	0
		I		0	0	0	0	0	0
		R		12.5%	93.33%	100%	50%	100%	100%
	CFM	S	—	87.5%	6.67%	33.34%	0	0	20%
		I		0	0	0	0	0	0
		R		12.5%	93.33%	66.66%	100%	100%	80%
Carbapenems	IMI	S	—	62.5%	13.33%	33.34%	50%	0	20%
		I		25%	6.6%	0	0	0	0
		R		12.5%	86.66%	66.66%	50%	100%	80%
	MER	S	—	87.5%	13.33%	66.66%	0	0	20%
		I		0	0	0	0	0	0
		R		12.5%	86.67%	33.34%	100%	100%	80%
B‐lactam combination agents	PTZ	S	—	62.5%	6.67%	0	50%	0	20%
		I		25%	0	0	0	0	0
		R		12.5%	93.33%	100%	50%	100%	80%

Abbreviations: Ak, amikacin; AZM, azithromycin; CAZ, ceftazidime; CFM, cefepime; CP, ciprofloxacin; CRO, ceftriaxone; CTX, cefotaxime; E, erythromycin; FOX, cefoxitin; Gm, gentamicin; I, intermediate; IMI, imipenem; LEV, levofloxacin; MER, meropenem; P, penicillin; PTZ, piperacillin tazobactam; R, resistant, based on CLSI guidelines; S, susceptible; SXT, trimethoprim/sulfamethoxazole; TE, tetracycline.

### 3.1. Viral Analysis

PCR products were sent for bidirectional sequencing and the sequencing results have been deposited in GenBank under Accession Numbers PQ577867, PQ577868, PQ577869, PQ577870, PQ577871, PQ577872, PQ577873, PQ577874, PQ577875, PQ577876, PQ577877, PQ577878, PQ577879, PQ577880, PQ577881, PQ577882, PQ577883.

The analysis of viral sequences involves aligning the sequences obtained from samples with those of reference strains and similar strains from various countries across different continents. This alignment is performed using the BioEdit software, which facilitates the comparison of genetic sequences. Following the alignment, phylogenetic trees are constructed to visualize the evolutionary relationships among the viral strains.

**Figure 3:**
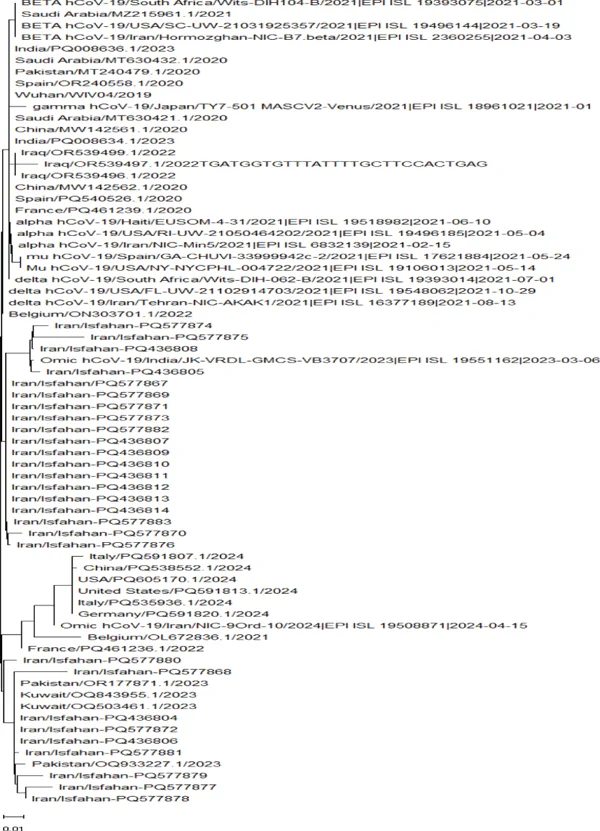
Phylogenetic tree of sequenced data and comparison with reference sequences.

**Figure 4:**
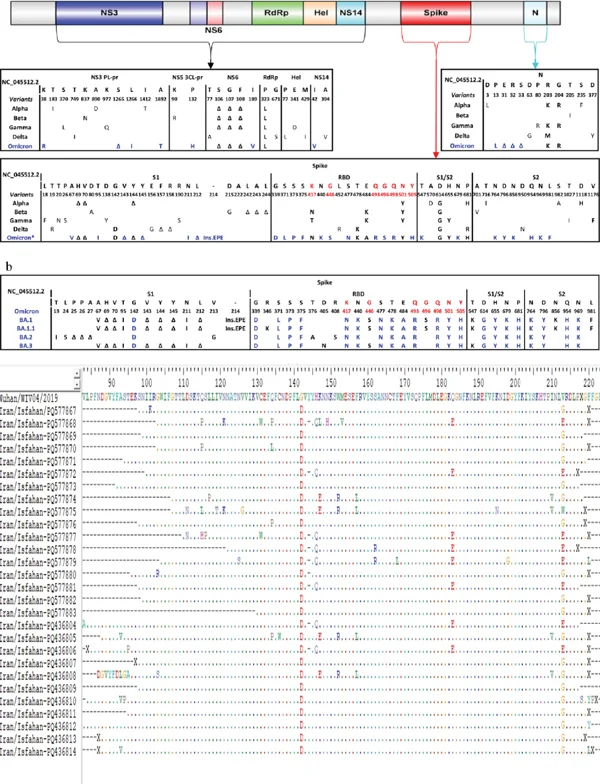
Bioinformatic analysis of SARS‐CoV‐2 spike protein variants.

To determine the types of variants in the collected samples, the sequences were uploaded to the GISAID database, where specific mutations associated with the variants were retrieved. Based on previous studies, the identification of mutations was facilitated by analyzing nucleotide positions and amino acid changes, which played a crucial role in variant classification. Genomic analysis of viral isolates identified Alpha, Delta, and Omicron variants, with hallmark spike mutations including *Δ*144 (Alpha) and G142D (Delta/Omicron) (Figures 3 and 4). Notably, rare substitutions such as Q183E and V213E were observed in a subset of samples, highlighting ongoing viral evolution (Table 2). Among the 53 patients with bacterial coinfection, *A. baumannii* was observed in 43.2% of Delta‐positive cases versus 18.8% of Alpha‐positive cases (95% CI for the difference: −0.5% to 49.3%). Due to the small sample size of the Alpha subgroup (*n* = 16), no inferential statistical testing was performed.

**Table 2:** The genetic variants identified in registered samples and comparative analysis with reference datasets.

Accession numbers	Mutation variant
PQ577867	I101K, G142D^$^, V213G
PQ577868	Q115P, N121K, C131W, Q134P, G142D^$^, Y144del^$^, H146Q^$^, K147L^$^, N149H^$^, M153V, Q183E, V213E, Q218R
PQ577869	G142D^$^, V213G
PQ577870	Q115P
PQ577871	G142D^$^, V213G
PQ577872	G142D^$^, Y144del^$^, H146Q^$^, Q183E, V213E
PQ577873	G142D^$^, V213G
PQ577874	L117P, G142D^$^, K147E^$^, W152R^$^, F157L, I210V, V213G
PQ577875	D111N, Q115L, I119T, N121K, V126G, G142D^$^, K147E^$^, W152R^$^, F157L, K195N, I210V, V213W
PQ577876	Q134P, G142D^$^, V213G
PQ577877	D111N, Q115H, S116P, C131W, G142D^$^, Y144del^$^, H146Q^$^, Q183E, V213E
PQ577878	G142D^$^, Y144del^$^, H146Q^$^, S162R
PQ577879	N125S, G142D^$^, Y144del^$^, H146Q^$^, S162R, F168L, Q183E, D198G, V213E
PQ577880	G103R, G142D^$^, Y144del^$^, V213G
PQ577881	G142D^$^, Y144del^$^, H146Q^$^, Q183E, V213E
PQ577882	G142D^$^, V213G
PQ577883	G142D^$^, V213G
PQ436804	G142D^$^, Y144del^$^, H146Q^$^, Q183E, V213E
PQ436805	A93V, Q134P, C136W, G142D^$^, K147E^$^, W152R^$^, F157L, I210V, V213G
PQ436806	T95P, G142D^$^, Y144del^$^, H146Q^$^, Q183E, V213E
PQ436807	G142D^$^, V213G
PQ436808	Ins92D, A93L, S94G, T95A, G103S, G142D^$^, K147E^$^, W152R^$^, F157L, I210V, V213G
PQ436809	G142D^$^, V213G
PQ436810	A93V, S94P, G142D^$^, V213G, Q218S, F220Y
PQ436811	G142D^$^, V213G
PQ436812	G142D^$^, V213G
PQ436813	G142D^$^, V213G
PQ436814	A93V, G142D^$^, V213G, F220Y

*Note:* The symbol "$" indicates that these mutations are associated with variants of concern (VOCs), including Alpha, Delta, and Omicron lineages.

In this study, the G142D mutation was identified in 26 samples. The concurrent presence of H146Q, Q183E, and V213E in several Alpha‐variant sequences raises the possibility of enhanced immune evasion. Analysis of spike‐protein mutations focused on the principal circulating lineages. The Alpha variant is characterized by the Y144del deletion (positions 141–146) within the N‐terminal domain (NTD), a change previously shown to reduce neutralization by multiple monoclonal antibodies; eight isolates in our cohort carried this deletion. G142D, a substitution shared by Delta and Omicron lineages and known to impair recognition by NTD‐directed antibodies, was the most frequent mutation detected. Two uncommon substitutions, Q183E and V213E (global prevalence < 0.01%), were also observed; their biological significance remains to be determined. Omicron‐related sequences displayed the expected combination of G142D with additional NTD deletions, further supporting the capacity of these alterations to facilitate antibody escape.

These observations highlight the value of continuous genomic monitoring to detect emerging spike mutations that may affect vaccine performance and the efficacy of therapeutic monoclonal antibodies.

## 4. Discussion

Our findings demonstrate a substantial burden of secondary bacterial and fungal infections among patients with severe viral respiratory disease, with nearly 40% of SARS‐CoV‐2–positive individuals developing coinfection. *K. pneumoniae* and *A. baumannii* predominated among the isolates, highlighting the prominent role of opportunistic gram‐negative pathogens in critically ill patients. These observations underscore the importance of systematic screening for bacterial coinfection in viral pneumonia, given its established contribution to disease progression and adverse clinical outcomes.

The coinfection rate observed in the present cohort was lower than the 94.2% reported by Zhu et al. [20], yet aligned with previous studies identifying *K. pneumoniae* and *A. baumannii* as the most frequent pathogens in COVID‐19 patients [21]. Such interstudy variation is most likely attributable to differences in geographic setting, intensive‐care practices, infection‐prevention capacity, and underlying patient characteristics. Collectively, our data support the wider recognition that nosocomial gram‐negative organisms particularly nonfermenting species constitute the predominant respiratory copathogens in critically ill individuals, independent of the primary viral etiology.

AMR patterns among the recovered isolates represent a major clinical concern. The high rates of carbapenem resistance observed in *A. baumannii* and *K. pneumoniae* mirror global trends reported from intensive‐care settings. In the present series, *A. baumannii* displayed near‐complete resistance to *β*‐lactams and carbapenems, severely limiting therapeutic options. Consequently, empirical antibiotic regimens are frequently suboptimal in coinfected patients, underscoring the necessity of routine susceptibility testing to enable targeted therapy and curb unnecessary antibiotic exposure. In contrast, *E. coli* isolates retained susceptibility to several agents, whereas *S. aureus* strains—although 50% were MRSA—remained fully susceptible to fluoroquinolones and macrolides. This marked heterogeneity in resistance profiles further complicates empirical decision‐making and reinforces the value of rapid diagnostics together with locally derived antibiograms for effective antimicrobial stewardship [22, 23].

Multiple factors are likely to have contributed to the high frequency of coinfection, including virus‐induced impairment of host immunity, prolonged mechanical ventilation, and intense antibiotic selective pressure during the pandemic, and temporary lapses in infection‐prevention measures during surges. These observations highlight the need to integrate robust antimicrobial stewardship programs with strengthened infection‐control practices, particularly within intensive‐care units where both selective pressure and opportunities for cross‐transmission are greatest.

As illustrated in Table 1, *A. baumannii* and *K. pneumoniae* exhibited resistance to nearly all tested agents, whereas *E. coli* and *S. aureus* (including 50% MRSA) retained broader susceptibility. Comparable high‐level resistance among *A. baumannii* isolates has been documented in several COVID‐19 cohorts worldwide. Bazaid et al. reported marked resistance to ceftriaxone, ceftazidime, trimethoprim–sulfamethoxazole, and gentamicin in sputum isolates from ICU patients [21]. Other investigations have similarly described near‐pan‐resistance in *A. baumannii* recovered from ETAs, with only colistin retaining partial activity [24].

Consistent with these observations, Sharifipour et al. found 100% meropenem resistance and 87.5% amikacin resistance among blood isolates of *A. baumannii* from critically ill COVID‐19 patients [25]. In an earlier Iranian study, Chan et al. likewise identified *A. baumannii* as the predominant copathogen (90% of cases), displaying extensive resistance to all agents tested except colistin; the few *S. aureus* isolates recovered included both MRSA and fully susceptible strains [26]. Collectively, these data reinforce the global challenge posed by multidrug‐resistant gram‐negative pathogens in COVID‐19–associated secondary infections.

In the present study, 50% of S. aureus isolates were MRSA, highlighting the added therapeutic complexity these organisms introduce in the intensive‐care setting. Prior work has shown that the risk of MRSA acquisition rises substantially with prolonged ICU stay, increasing 2.5‐ to 4‐fold when hospitalization exceeds 1 week [26]. The widespread empirical use of antibiotics during the COVID‐19 pandemic is likely to have intensified selective pressure, facilitating the emergence and dissemination of multidrug‐resistant strains [15]. Concurrently, the predominance of variants of concern (VOCs) particularly Delta reinforces the value of rapid, sequencing‐based surveillance to track changes in viral pathogenicity and immune‐escape potential.

Genomic analysis of circulating SARS‐CoV‐2 strains confirmed the predominance of the Delta variant, which, together with Alpha, belonged to recognized VOCs. Although viral evolution was not the primary focus of this investigation, the findings underscore the value of integrating genomic surveillance with routine clinical microbiology. Among sequenced cases, Delta (69.8%) was associated with a higher frequency of *A. baumannii* coinfection than Alpha; however, the limited number of Alpha cases precluded formal statistical comparison. No consistent relationship was identified between individual spike mutations (e.g., G142D or Q183E) and the AMR profiles of coinfecting bacteria. These preliminary observations highlight the need for larger prospective studies to determine whether viral genotype influences the risk or spectrum of secondary bacterial infection. Viral evolution may modulate host–pathogen interactions, immune responses, and patterns of hospitalization, thereby indirectly shaping susceptibility to bacterial superinfection. Regional surveillance data from Iran support the temporal dynamics observed here. Mahase documented the progressive appearance of the N501Y substitution from December 2020, marking the introduction of Alpha, followed by the co‐occurrence of L452R and T478K mutations from May 2021 that signaled the subsequent dominance of Delta in Khuzestan province [16]. The rapid global dissemination of successive VOCs further illustrates the capacity of key spike mutations, such as N501Y, to enhance immune escape and reduce neutralization by antibodies raised against the ancestral Wuhan‐Hu‐1 strain [19, 27, 28].

This study offers several strengths, including the integration of microbiological, genomic, and antibiotic susceptibility data over a 2‐year period. However, limitations must be acknowledged. First, the study population consisted exclusively of COVID‐19 patients, limiting the generalizability of findings to other viral respiratory illnesses such as influenza. Second, culture‐based methods may underestimate fastidious or difficult‐to‐culture organisms. Third, the single‐center design limits external validity. Multicenter, longitudinal studies are needed to determine how viral evolution, healthcare practices, and antibiotic use interact to shape coinfection patterns globally.

## 5. Conclusion

Bacterial coinfections significantly contribute to morbidity in severe viral respiratory diseases. The predominance of multidrug‐resistant *A. baumannii* and *K. pneumoniae* in our ICU setting suggests that empirical carbapenems may be ineffective; instead, routine susceptibility testing and targeted therapy based on local antibiograms are essential. During future respiratory pandemics, ICUs should integrate rapid bacterial diagnostics with antimicrobial stewardship to limit inappropriate antibiotic use. Furthermore, combining viral genomic surveillance with bacteriological monitoring provides a comprehensive framework for patient management and outbreak preparedness.

## Author Contributions

S.J.: data curation, formal analysis, investigation, software, visualization, writing—original draft, writing—review and editing. F.M.R.: methodology, writing—original draft, writing—review and editing. B.N.E.: funding acquisition, resources, supervision, validation, writing—review and editing. S.M., K.S., and E.N.: supervision, review, and editing.

## Funding

This study was supported by Isfahan University of Medical Sciences (10.13039/501100003970, 3400945).

## Ethics Statement

Ethical approval for this study was obtained from the Ethics Committee of Isfahan University of Medical Sciences (Approval ID: IR.MUI.MED.REC.1400.839). Since this was a retrospective laboratory‐based study using anonymized residual specimens, registration in a clinical trial registry was not applicable.

## Consent

The authors have nothing to report.

## Conflicts of Interest

The authors declare no conflicts of interest.

## Data Availability

The datasets generated and analyzed during the current study are not publicly available but are available from the corresponding author on reasonable request. The nucleotide sequences generated during this study are available in the GenBank repository under Accession Numbers PQ577867, PQ577868, PQ577869, PQ577870, PQ577871, PQ577872, PQ577873, PQ577874, PQ577875, PQ577876, PQ577877, PQ577878, PQ577879, PQ577880, PQ577881, PQ577882, and PQ577883.
